# Diet quality in Brazilian adolescents with cystic fibrosis

**DOI:** 10.1016/j.jped.2025.101488

**Published:** 2025-12-23

**Authors:** Chiara Pascon, Maria Angela Bellomo-Brandão, José Dirceu Ribeiro, Elizete Aparecida Lomazi

**Affiliations:** a Universidade Estadual de Campinas, Faculdade de Ciências Médicas, Programa de Pós-Graduação em Saúde da Criança e do Adolescente, Campinas, SP, Brazil; b Universidade Estadual de Campinas, Faculdade de Ciências Médicas, Departamento de Pediatria, Campinas, SP, Brazil

**Keywords:** Diet, Food and nutrition, Adolescent nutrition, Nutritional status, Cystic fibrosis, Feeding behavior

## Abstract

**Objective:**

To describe the quality of the diet and its relationship with lung function and nutritional status in adolescents with cystic fibrosis (CF).

**Methods:**

A questionnaire was applied to 47 adolescents (12–19 years old) followed at a university reference center. Lung function impairment was classified as mild (FEV1pp ≥ 60%), moderate (FEV1pp 41–59%), or severe (FEV1pp < 40%) using spirometry. Food consumption frequency was classified as rarely, 1-2 times/week, 3-4 times/week, or ≥ 5 times/week. Linear regression analysis was used to model the relationship between lung function, BMI/age Z-score, and food frequency. Principal component analysis (PCA) was performed to identify patterns of diet quality.

**Results:**

The mean BMI/age Z-score was -0.62. Approximately 60% of adolescents had FEV1 ≥ 60%, and 21% had severe impairment (FEV1 < 40%). Better BMI/age Z-score was observed in groups with higher consumption frequencies of vegetables and fruit (≥ 5/week), white meat (> 3-4/week), and oil (> 3-4/week). Linear regression models identified significant relationships: BMI/age Z-Score = -3.25+ 0.8182*meat_b+0.5082*vegetables_fruit (p < 0.001): Increased white meat, fruit, and vegetable consumption positively influenced the BMI/age Z-Score; lung function = 0.6927 + 0.3356*vegetables-0.2406*fruit_vegetables-0.4550*fast_food (p < 0.001): Lower consumption of fruit and vegetables and higher fast-food intake were associated with more severe lung function; and lung function = 0.39376 -0.32345 *ZSCORE (p < 0.0001): The BMI/age Z-Score positively influenced lung function. PCA confirmed the positive relationship between fruit, vegetables, and white meat consumption and BMI/age Z-score.

**Conclusion:**

Higher consumption of fruit, vegetables, and meat has a positive influence on the BMI/age Z-score and lung function among adolescents with CF.

## Introduction

Cystic fibrosis (CF) is an autosomal recessive disease resulting from mutations in the gene that encodes the Cystic Fibrosis Transmembrane Conductance Regulator (CFTR) protein. CFTR function deficiency leads to the production of thick and abnormal mucous secretions, causing a systemic chronic disease with primary manifestations related to respiratory and gastrointestinal (GI) symptoms. GI symptoms are now recognized as a major priority, extending beyond pancreatic insufficiency [1,2]. Thick mucus in the respiratory tract creates an environment conducive to bacterial colonization, generating an inflammatory and infectious condition that consumes energy and contributes to a loss of lung function, chronic malnutrition, and reduced life expectancy [3].

While studies investigating the isolated effects of nutrients were essential, they were insufficient to demonstrate the relationship between food and health. Analyzing food consumption involves more than simply quantifying nutrient intake; it requires examining the diet as a whole, considering sociodemographic characteristics and behaviors, which would therefore benefit the approach, evaluation, and reinforcement of behaviors during nutritional treatment [4].

To the best of our knowledge, no Brazilian study has examined dietary patterns in adolescents with CF. Hence, the aim of this proposal is to provide a clearer understanding of the factors that may influence the nutritional status of this population.

## Methods

This was a prospective observational, cross-sectional, and primary study. A survey on food consumption was applied to a convenience sample of 47 adolescents aged 12 to 19 years who were followed at a Brazilian Reference Center for Cystic Fibrosis. The CF diagnosis was confirmed by genetic testing and/or sweat testing. The study included all CF patients followed at the reference center in the defined age range who were not receiving CFTR modulators.

Informed consent and participant assent were obtained prior to participation in the study. Participants were invited to a face-to-face interview during their medical visit at the reference outpatient clinic between 2021 and 2022. Anthropometric measurements were taken according to standard procedures. BMI and BMI/age Z-score were calculated using the WHO ANTHRO software [5].

The questionnaire also included data on school type (public or private), eating frequency, screen use during meals, and physical activity. Food consumption was evaluated using a Food Frequency Questionnaire (FFQ). Parents/caregivers assisted with the responses to ensure greater reliability and accuracy. The FFQ is composed of groups based on research by Dishchekenian et al., and their food items were adapted to reflect the local population [6]. The categories of food intake frequency were: Rarely; 1–2 times per week; 3–4 times per week; and ≥ 5 times per week.

To analyze the psychometric characteristics of the scales, reliability analyses were performed using Cronbach's alpha. The alpha value ranges from 0 to 1, with the following readings: greater than 0.9: very good consistency; between 0.8 and 0.9: good; between 0.7 and 0.8: reasonable; between 0.6 and 0.7: poor; and less than 0.6: unacceptable. Cronbach's alpha was performed for the dietary profile variables (meals_at_home, meals_away_from_home, who_prepares_meals, meals_per_day, screen_time_during_meals, physical_activity) and food consumption (rice_pasta, oil, vegetables, red_meat, sausages, sweets, white_meat, sugar, butter_margarine, vegetables_fruit, eggs, bread, cow's milk, juice, cookies_sweets, hamburgers, soft drinks, chocolate, fast_food, mayonnaise).

According to the Reference Center care protocol, patients undergo annual spirometry. The most recent evaluation prior to the interview was recorded. Lung function severity was rated as follows: FEV1pp ≥ 60 % indicates mild respiratory functional loss, FEV1pp from 41 % up to 59 % indicates moderate loss and FEV1pp<40 % indicates severe loss.

A Kruskal–Wallis analysis followed by a *post hoc* test was performed to assess the relationship between lung function and BMI/age Z-score.

Linear regression models were conducted to further explore the variables and their interactions. The p-value of the F test was reported; when significant, the linear regression model and its respective p-value were presented.

A Principal Component Analysis (PCA) was performed to study food consumption profiles, followed by a cluster analysis based on the PCA results.

A significance level of 0.05 was used for all statistical analyses, and the statistical program R version 4.2.1 (Funny Looking Kid) was used, along with the packages factoextra, missMDA and FactoMineR [7, 8, 9].

All procedures were approved by the institution's ethics committee, CAAE 20,242,719.7.0000.5404.

## Results

The distribution of clinical and lifestyle factors, as well as behavior during a specific meal, as reported by 47 adolescents with Cystic Fibrosis (CF), is presented in Table 1.

**Table 1:** Distribution of clinical, lifestyle characteristics and behavior during a specific meal, as reported by 47 adolescents with cystic fibrosis.

CHARACTERISTICS	
AGE (YEARS)	**Mean ± SD [CI]**
	16.5 ± 3.2 [15.6 – 17.5]
BMI/AGE Z-SCORE	- 0.62 ± 1.45 [−1.05; −0.19]
SEX	**N (%)**
MALE	25 (53.2)
FEMALE	22 (48.6)
SEVERITY OF PULMONAR FUNCTION	
MILD	29 (61.7)
MODERATE	8 (17)
SEVERE	10 (21.3)
SCHOOL	
PUBLIC	30 (64)
PRIVATE	17 (36)
PRACTICING PHYSICAL ACTIVITY	
YES	34 (71.8)
NO	13 (28.2)
PHYSICAL ACTIVITY/WEEK⁎⁎⁎	
RARELY	2 (5.9)
1–2	15 (44.1)
3–4	11(32.3)
≥5	6 (17.4)
MEALS AT HOME/WEEK	
RARELY	0
1–2	0
3–4	8(17)
≥ 5	39 (83)
COOK MEALS	
PARENT/GUARDIAN	40 (85.1)
PARTICIPANT	5 (10.6)
BOTH	2 (4.3)
NUMBER OF MEALS PER DAY	
2	1 (2)
3	1(2)
4	9(19)
5	16(34)
6	20(42)
SCREEN USE DURING MEAL	
YES	35 (74)
NO	12 (26)

BMI, Body Mass Index; SD, standard deviation; CI, confidence interval; N, Number.

⁎⁎⁎ 34 participants practice physical activity.

Food consumption groups indicated a higher frequency, 5 or more times a week, for the rice and pasta food group and for the oil and milk, and dairy products group.

Eighteen participants (38 %) reported consuming red meat at least five times a week, while 10 (21 %) reported consuming white meat with the same frequency. Egg intake was less common, as 34 of 47 adolescents reported consuming them rarely or 1–2/week.

Table 2 presents the associations between BMI/age Z-score values, lung function, sociodemographic characteristics and eating behaviors. Food groups showing a significant association with the Z-score included vegetables and fruit, white meat and oils; higher consumption of these groups was associated with higher BMI/age Z-score.

**Table 2:** Relationship between BMI/age Z-score and lung function, sociodemographic characteristics, and food consumption frequency among adolescents with CF (*n* = 47). A p-value of less than 0.05 indicates that at least one of the studied factors influences the BMI/age- Z-score or lung function. Categories that differ from each other are shown in parentheses.

	p-value Z-score (BMI/age)	p-value lung function
Sex	0.993	0.377
Type of school	0.648	0.285
Practice of physical activity	0.496	0.749
Physical activity frequency	0.145	0.239
Meals at home frequency	0.699	0.690
Meals out of home	0.341	0.097
Cook meals	0.152	0.144
Number of meals per day	0.917	0.890
Screen use per meal	0.637	0.420
CONSUMPTION FREQUENCY	0.993	0.377
Rice and pasta	0.815	0.528
Bread	0.628	0.751
Legumes	0.932	0.071
Vegetables and fruits	0.011 (0–3)^1^	0.194
Eggs	0.752	0.669
Milk and derivatives	0.275	0.049
Red meat	0.238	0.886
Sausages	0.921	0.408
White meat	< 0.001 (0–2, 0–3, 1–2, 1–3)^2^	0.080
Sweets	0.05 (0–3, 1–3)^3^	0.089
Sugar	0.3	0.384
Cookies, cakes or pie	0.129	0.011*
Butter/ margarine	0.315	0.584
Oils	0.010 (2–1, 3–1)^4^	0.472
Juices	0.348	0.255
Hamburguer	0.808	0.806
Soda	0.761	0.820
Chocolate	0.761	0.974
Fast food	0.216	0.090
Mayonnaise	0.985	0.056

p-value < 0,05; Kruskal Wallis Test; BMI, Body Massa Index; Categories: 0 = rarely; 1 = 1–2 times a week; 2 = 3- 4 times a week; 3 = 5 or more times a week.

1 Significant difference found only between the frequencies of consuming rarely versus consuming vegetables and fruits 5 or more times per week.

2 Significant difference found whenever white meat consumption exceeded 3 to 4 times per week.

3 Significant differences found only when consumption of sweets exceeded 5 or more times per week.

4 Significant differences found only when consumption of oil exceeded 3 to 4 times per week.

A Kruskal–Wallis analysis was performed in a bivariate study to verify whether there was a significant relationship between pulmonary function and Z-scores. This was followed by a *post hoc* test, which revealed a significant difference in Z-scores between participants with severe and mild pulmonary function loss. Statistically, patients with mildly impaired lung function had higher Z-scores than those with severe impairment.

A regression analysis was conducted to study the relationship between food consumption and eating behaviors. These behaviors included location of meals (at home or away), person responsible for meal preparation, number of meals per day, and screen use during meals. None of the regressions was significant.

Linear regression analysis allowed equations to be constructed to establish relationships between food consumption frequency and nutritional status, assessed by BMI/age Z-score. Initially, a regression was performed with the food consumption variables [Z-Score ∼ rice_pasta + oil + vegetables + meat_*v* + sausages + sweets + meat_*b* + sugar + butter_marg + vegetables_fruit + eggs + bread + milk_*d* + juice + cookies_sweets + hamburgers + soft drinks + chocolate + fast food + mayonnaise]. In this case, the BMI/age Z-score had no significant relationship with the variables, with an F-test p-value of 0.08. However, subsequent linear regressions excluding some variables identified the following model: Z-Score = −3.25+ 0.8182*meat_*b* + 0.5082*vegetables_fruit (*p* < 0.001), Alpha = −3.25, beta 0.8182. In this model, white meat intake had a positive effect of 0.81, and vegetable and fruit intake had an effect of approximately 0.51. The consumption of vegetables, fruits, and white meat was found to positively influence the BMI/age Z-Score. In the linear regression of lung function with food consumption frequency variables, the p-value of the F-test was 0.1519. [lung_function ∼ rice_pasta + oil + vegetables + meat_*v* + sausages + sweets + meat_*b* + sugar + margarine + vegetables_fruit + eggs + bread + milk_*d* + juice + cookies_sweets + hamburgers + soda + chocolate + fast food + mayonnaise]. After removing some variables, new linear regressions were performed, and the resulting model was lung_function = 0.6927 + 0.3356*vegetables-0.2406*fruit_vegetables-0.4550*fast_food (*p* < 0.001). Thus, the results indicate that individuals who frequently consume vegetables have their lung function improved by 0.33, while individuals who consume a combination of vegetables and fruits have their lung function increased by 0.24, and individuals who consume more fast food also have their lung function reduced by 0.45, i.e., the quality of the diet is lower in participants with more severe lung function impairment. Linear regression analysis also evaluated the relationship between lung function and BMI/age Z-score. The model lung function = 0.39376 −0.32345 *ZSCORE (*p* < 0.0001), indicated that an increase of 1 point in the Z-Score is associated with an increase of 0.32 in the Lung Function value.

### Psychometric analysis of the frequency questionnaire

The authors obtained a Cronbach's alpha value of 0.667, which measures the internal consistency of an assessment instrument. Values between 0.6 and 0.8 are considered acceptable.

### Principal components analysis (PCA)

**Figure 1:**
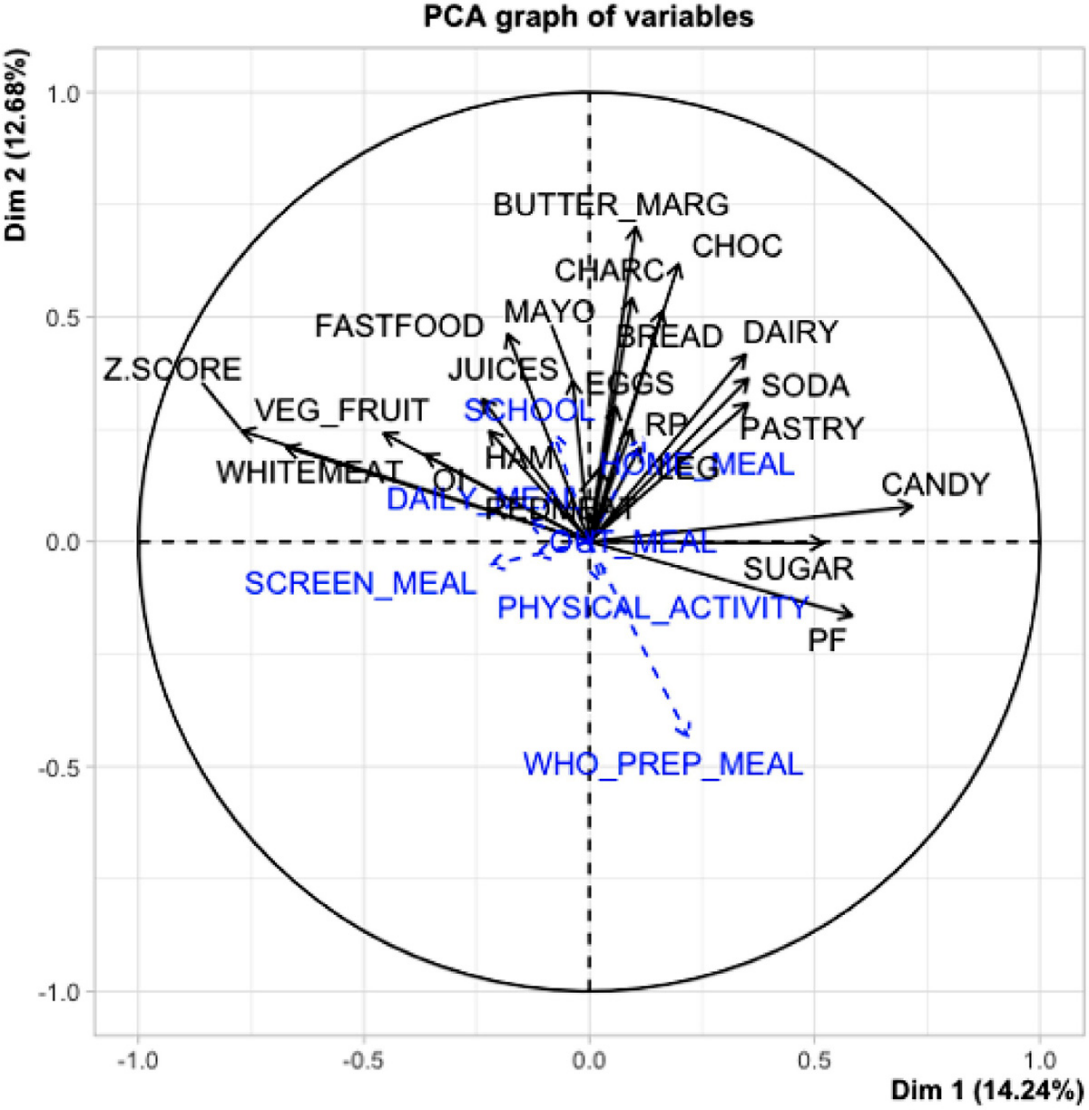
PCA plot of food consumption variables as the main variable, and dietary profile as a supplementary variable in relation to the Z-Score and PF. Dim, dimension; RP, rice and pasta; OI, oils; LEG, legumes; REDMEAT, red meat; EMBU, sausages; CANDY; WHITEMEAT, white meat; SUGAR; BUTTER_MARG, butter/ margarine; VEG_FRUIT, vegetables and fruits; EGGS; BREAD; DAIRY, milk and derivatives; JUICES; PASTRY, cookies, cakes or pies; HAM, hamburguer; SODA; CHOC, chocolate; FASTFOOD; MAYO, mayonnaise; Z.SCORE; SCREEN_MEAL, screen use during meals; WHO_PREP_MEAL, who prepares the meal; PF, lung function; SCHOOL, education; HOME_MEAL, meals eaten at home; OUT_MEAL, meals eaten out of home; PHYSICAL_ACTIVITY, practice of physical activity; DAILY_MEAL, number of daily meals.

**Figure 2:**
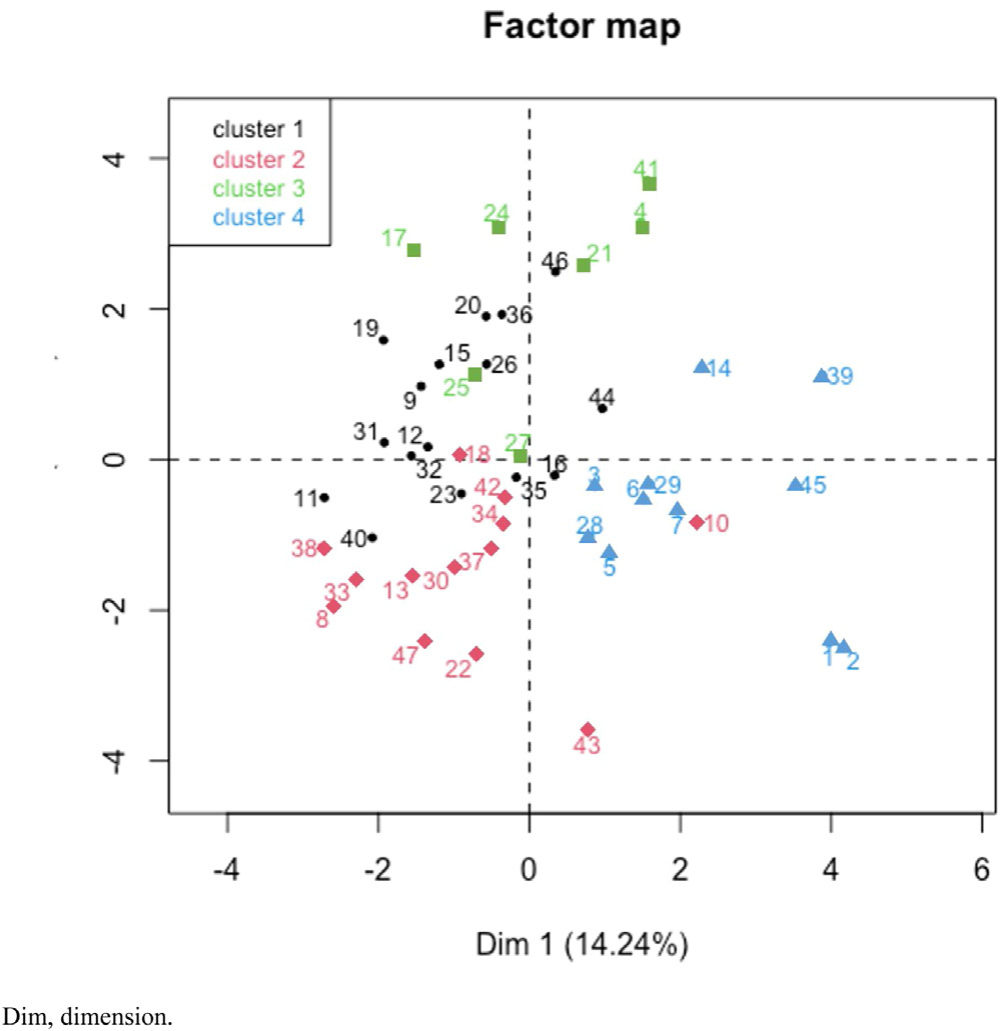
Graphic representation of the position of participants in the PCA with food consumption as the main variable, and dietary profile as a supplementary variable in relation to BMI/age Z Score and pulmonary function. Dim, dimension.

PCA revealed a positive and statistically significant relationship between BMI/age Z-score and the consumption of white meat, vegetables, and fruit. No statistically significant relationship was identified between consumption of sugar/sweets and BMI/age Z-score (Figure 1). Cluster analysis revealed four groups defined by their predominant food consumption patterns. Three of these groups had a higher proportion of children classified as eutrophic who frequently consumed protein sources such as meat, milk, or dairy products. A fourth group, characterized by poorer lung function and lower Z-scores, showed higher consumption of sugar, bread, milk and dairy products, and lower intake of white meat, eggs, vegetables and fruit (Figure 2).

## Discussion

The present data indicate that consuming fruits, vegetables, and meat has a positive influence on the nutritional status and lung function of adolescents with CF.

Poor lung function, hypoxemia, and chronic inflammation can cause gastrointestinal symptoms and loss of appetite in patients with CF [10]. The association between lung function and nutritional status has been recognized in these patients. This study contributes to this knowledge by observing a group of adolescents and evaluating the association between poor diet quality and disease severity. Disease severity was evaluated based on poor nutritional status and poor lung function. This broadens the health team's understanding of the relationship between diet and disease, creating new opportunities for intervention beyond providing guidance on the consumption of micronutrients and macronutrients.

According to the WHO classification, the mean BMI/age Z-score of −0.62 found in the group of adolescents included in this study indicates adequate nutritional status [5]. This positive outcome is primarily due to the professional care provided at the Reference Center. Professionals collaborate to focus on patients' nutritional status, ensuring an interdisciplinary approach to care. More than half of the adolescents in the study group had relatively preserved lung function (FEV1pp ≥ 60 %), indicating that the severity of the disease is predominantly mild in this cohort [4].

The general data indicate that adolescents consume fruit and vegetables more frequently than fast food. This positive food consumption profile may be related to the fact that two-thirds of patients attended public schools. Public schools in Brazil have regulations concerning the sale of ultra-processed foods in school cafeterias and also offer balanced, government-funded meals [11]. A Turkish study found that adolescents attending public schools were less likely to prefer healthy foods for snacks compared to those attending private schools. Socioeconomic status has been recognized as a relevant factor affecting dietary habits in adolescence. Considering that income levels vary considerably between adolescents in private and public schools, the increased consumption of snacks by those attending private schools may be related to greater financial resources [12].

Cluster analysis indicated that groups with the most suitable nutritional profiles had the greatest consumption of protein sources such as milk and dairy products, as well as white and red meat. Adolescents with the poorest nutritional status and lung function values reported lower protein consumption than those with better lung function. Those with poorer lung function tended to consume bread, sugar, milk and dairy, which may suggest poor dietary quality. The literature also describes that patients with severe loss of lung function consume a smaller amount of food. In a vicious cycle, malnutrition worsens lung function, and the deterioration of lung function reduces food consumption [13]. Hypoxia regulates metabolism at several levels, for example, through mitochondrial ATP production, glucose uptake, and glycolysis. Therefore, it is likely that hypoxia also affects the production and/or action of many peptide hormones linked to food intake and appetite control [14]. The relationship between lung function decline and nutritional status associates disease severity and elevated serum leptin levels in patients with cystic fibrosis [15]. Leptin is an anorexigenic peptide that plays an important role in regulating food intake and energy expenditure by increasing energy expenditure and decreasing food intake [16].

Although the present study is limited by the small sample size, the results are consistent with the characteristic food intake of patients with chronic hypoxia: a preference for sweet, low-protein foods. This was also demonstrated in a randomized controlled trial involving people in a hypoxic state [17].

Sociodemographic and lifestyle factors influence diet quality; therefore, the results obtained in this study cannot be generalized to other populations. Education, income level, and geography are all interfering factors. These factors interact in complex ways, affecting not only food selection but also eating behavior and cultural practices. Hence, when addressing nutrition issues, it is essential to consider these sociodemographic aspects to promote more effective and targeted interventions. Psychosocial and cultural factors also affect the optimization of nutritional support, including clinical manifestations and disease severity, caregiver education and income, and access to care [18].

The NOVA food classification defines ultra-processed foods (UPFs) as industrial formulations made from food-derived substances that have been modified and recombined with multiple additives. While not all takeaway foods are ultra-processed, most of those commonly offered by fast food outlets and delivery services are largely composed of UPFs [19]. Additionally, under the best sociodemographic conditions for nutritional support, patients with cystic fibrosis tend to consume ultra-processed foods, mirroring the trend observed in the general population [20].

Newer therapies, such as CFTR modulators, have played a significant role in improving the nutritional status of patients with CF [21]. As a result of CFTR modulators, cystic fibrosis is becoming an increasingly prevalent disease in adults who face new nutritional challenges, including obesity [22]. As modulators become a key part of management, the present study provides data on the natural course of CF and allows for comparison with future studies.

Regarding the nutritional assessment tool, the authors must address an emerging issue. Contrary to the present findings, the paradigm linking poor nutritional status, as assessed by anthropometric measurements, and impaired lung function in patients with CF has recently been challenged by a retrospective, mixed cross-sectional and serial measures study including Australian children with CF (8 – 18 years) attending Sydney Children's Hospital (2007–2020). Body composition measures, including fat-free mass index FFMI and fat mass index (FMI) were taken from biennial dual energy x-ray absorptiometry (DXA) scans. Repeated correlation analyses found a weak positive correlation between FFMI-z, FMI-z, and BMI with FEV1pp, which led to the conclusion that nutritional status may be less influential on lung function than previously supposed [23]. These results may indicate a multifactorial causal relationship between body composition and lung function, which would be a significant finding.

Although the authors have conducted a prospective study, they recognize several limitations, including data collection at a single reference center, a limited sample size, and the use of a food frequency questionnaire that did not include a three-day recall, as is standard in other food consumption studies. Nevertheless, despite these limitations, the authors obtained meaningful results in the context of the pathophysiology and sociodemographic aspects of the patients. Despite the small sample size, a differentiated statistical analysis yielded significant findings.

The authors concluded that consumption of a diet recognized as healthy by adolescents with CF and monitored at a referral center had positive effects on lung function and BMI.

Despite the small size of the cohort, it was possible to evaluate PCA and identify the eating habits of adolescents with CF in a practical way, using a questionnaire, as well as their relationship with the severity of the disease. The present results could potentially be useful for assessing and providing dietary guidance to Brazilian adolescents with CF.

Brazilian children referred to the reference center with deteriorated lung function presented poor food preference quality. During treatment, it is essential to educate parents about the importance of a nutritious diet for their child. It is crucial to emphasize the long-term benefits of establishing healthy eating habits and to provide effective strategies to help overcome any challenges related to dietary intake during this period.

## Data availability statement

The data that support the findings of this study are available from the corresponding author.

## Conflicts of interest

The authors declare no conflicts of interest.
